# Bacterial Flora Changes in Conjunctiva of Rats with Streptozotocin-Induced Type I Diabetes

**DOI:** 10.1371/journal.pone.0133021

**Published:** 2015-07-15

**Authors:** Chao Yang, Yuda Fei, Yali Qin, Dan Luo, Shufei Yang, Xinyun Kou, Yingxin Zi, Tingting Deng, Ming Jin

**Affiliations:** 1 Sino-Japan hospital, No. 1, Yinghua East Street, Beijing 100029, China; 2 Beijing Hospital of Traditional Chinese Medicine (TCM), Beijing 100010, China; 3 Eye Hospital, China Academy of Chinese Medical Sciences, Beijing 100040, China; Beijing Institute of Microbiology and Epidemiology, CHINA

## Abstract

**Background:**

The microbiota of both humans and animals plays an important role in their health and the development of disease. Therefore, the bacterial flora of the conjunctiva may also be associated with some diseases. However, there are no reports on the alteration of bacterial flora in conjunctiva of diabetic rats in the literature. Therefore, we investigated the changes in bacterial flora in bulbar conjunctiva of rats with streptozotocin (STZ)-induced type I diabetes.

**Methods:**

A high dose of STZ (60 mg/kg, i.p.) was injected into Sprague-Dawley (SD) rats to induce type I diabetes mellitus (T1DM). The diabetic rats were raised in the animal laboratory and at 8 months post-injection of STZ swab samples were taken from the bulbar conjunctiva for cultivation of aerobic bacteria. The bacterial isolates were identified by Gram staining and biochemical features. The identified bacteria from both diabetic and healthy rats were then compared.

**Results:**

The diabetic and healthy rats had different bacterial flora present in their bulbar conjunctiva. In total, 10 and 8 bacterial species were found in the STZ and control groups, respectively, with only three species (*Enterococcus faecium*, *Enterococcus gallinarum* and *Escherichia coli*) shared between the two groups. Gram-positive bacteria were common in both groups and the most abundant was *Enterococcus faecium*. However, after the development of T1DM, the bacterial flora in the rat bulbar conjunctiva changed considerably, with a reduced complexity evident.

**Conclusions:**

STZ-induced diabetes caused alterations of bacterial flora in the bulbar conjunctiva in rats, with some bacterial species disappearing and others emerging. Our results indicate that the conjunctival bacterial flora in diabetic humans should be surveyed for potential diagnostic markers or countermeasures to prevent eye infections in T1DM patients.

## Introduction

Diabetes mellitus (DM) is ranked 15^th^ worldwide for diseases causing YLL (Years of Life Lost), accounting for approximately 2% of the total YLL in 2012, according to the World Health Organization (http://apps.who.int/iris/bitstream/10665/112738/1/9789240692671_eng.pdf?ua=1). Diabetes is a complex metabolic disorder influenced by multiple factors, including the environment and genetics. Recently, the intestinal microbiota in humans has been suggested to play an important role in diabetes development by interacting with environmental parameters and susceptible genetic factors [1]. Microbiota in other parts of human body, including oral, skin, conjunctival, vaginal and respiratory microbiota, also play roles in both human health and disease development [2]. Associations between type I/II diabetes mellitus (T1DM/T2DM) and intestinal or oral microbiota have previously been demonstrated [3–6]. Recently, the probiotic bacterium *Lactobacillus casei* Zhang was confirmed to be able to reduce the susceptibility of Sprague-Dawley (SD) rats to type 2 diabetes by regulating microbiota-mediated chloride ion influx [7].

The diversity of bacterial flora in human conjunctiva was revealed by high throughput deep sequencing of 16S rDNA amplified from ocular surface swab samples [8]. There were 59 bacterial genera in all four of the tested healthy human conjunctiva, and 12 of them, namely *Pseudomonas*, *Propionibacterium*, *Bradyrhizobium*, *Corynebacterium*, *Acinetobacter*, *Brevundimonas*, *Staphylococci*, *Aquabacterium*, *Sphingomonas*, *Streptococcus*, *Streptophyta* and *Methylobacterium*, are core microbiome, accounting for >96% of bacteria [8]. The conjunctival microbiota is altered after death [9], antibiotic treatment [10], and trachoma infection [11]. In an experimental animal model, the bacterial flora in rabbit conjunctiva was reduced after administration of fresh onion juice [12]. Research reports on bacterial flora in rats are rare. In this study, we used a streptozotocin (STZ)-induced diabetes model in rats for investigating alterations in the conjunctival bacterial flora due to diabetes relative to healthy rats.

The STZ-induced diabetic rat model is widely used in diabetes research [13–17]. STZ is a diabetogenic agent that selectively destroys pancreatic beta-cells. Both T1DM and T2DM can be induced, by a high dose (40 to 60 mg/kg body weight) and low dose (35 mg/kg body weight) of STZ, respectively [18]. In this study we used a high dose of STZ to induce T1DM in SD rats for comparison of bacterial flora changes in conjunctiva.

## Materials and Methods

### Animals used in this study

Six-week-old male Sprague-Dawley (SD) rats with a body weight of 200–240 g were purchased from Huafukang Biotechnology Co. Ltd. (permission certificate no. SCXK (Beijing, China) 2014–0004). The animals were housed in the animal center of our hospital. This study was approved by the ethics committee of the Sino-Japan Hospital, Beijing, China. All animal experiments were performed humanely according to *Beijing Administration Rule of Laboratory Animal*.

### Induction of type 1 diabetes in rats

Twelve SD rats were randomly divided into two groups, STZ-induced type I diabetes group (STZ group, n = 7) and control group (n = 5). Each rat in the STZ group was injected with a single dose of 60 mg/kg of STZ dissolved in sodium citrate buffer pH 4.5 i.p. [19]. The control group was injected with the same amounts of sterile normal saline i.p. Rats in the STZ group were housed in separate cages while the rats in the control group were housed in a single large cage. Food and water were available *ad libitum*. Before blood glucose levels were determined, the rats were fasted overnight and then blood was taken from the tail vein. Rats with blood glucose levels exceeding 16.7 mmol on 7 consecutive days were considered diabetic and used in this study.

### Bacterial isolation and identification

Conjunctival swabs were collected from both eyes of the rats, in both groups, 8 months post-STZ injection. Sterile cotton swabs were wiped along the upper bulbar conjunctiva of both eyes by everting the eyelid [12]. During sampling, care was taken to avoid any contamination of the swabs by contact with the eyelid skin, eyelashes or lacrimal punctum. The swabs were immediately put into Luria-Bertani (LB) broth and the broth was incubated at 37°C overnight. After approximately 24 hours, 0.5 ml of the cultures were plated on to LB agar plates for isolating bacteria. After a further approximately 24 hours of growth at 37°C, colonies of different appearance were picked from each plate for sub-cultivating two times to purify the bacterial colonies. The colonies were identified by Gram staining and biological features using an automated microbiology system, VITEK 2 (bioMérieux Inc., Hazelwood, MO, USA).

## Results and Discussion

The microbiota of both humans and animals is important for their health and also plays key roles in disease development [1, 7, 20–22]. Moreover, STZ-induced T1DM and T2DM rat models are commonly used for research on diabetes. However, there is still no investigation of the bacterial flora changes in conjunctiva of diabetic rats. In this study, we induced T1DM in SD rats to study the bacterial flora changes present in bulbar conjunctiva using conventional isolation of aerobic bacteria techniques.

Diabetes was successfully induced by STZ as the blood sugar in the STZ group was significantly higher than the control group (*P* < 0.05) and typical diabetes symptoms, including polydipsia, polyphagia, polyuria, emaciation, weakness, obviously matte fur and hunchbacked, were developed.

The overnight growth of the LB broth inoculated with the swab samples became turbid, and after inoculating the overnight cultures onto LB agar plates for approximately 24 hours at 37°C, colonies could be observed on the plates. However, to our surprise, the appearance of the colonies on each plate was always the same. We had expected to observe many colonies with different shapes, sizes or colors. Therefore, we selected four colonies from each plate doing our best to choose those with slight different appearances. The bacterial colonies were then sub-cultivated two times in order to purify the colonies. The colonies in the third subcultures were used for further identification by Gram staining and biological characteristics using an automated microbiology system.

The bacterial identification results are shown in Table 1, and indicate that the diabetic and healthy rats have different bacterial flora present in their bulbar conjunctiva. In total, 10 and 8 bacterial species were found in the STZ and control groups, respectively, with only three species (*Enterococcus faecium*, *Enterococcus gallinarum* and *Escherichia coli*) shared between the two groups. Gram-positive bacteria are common to both groups, with the most abundant species being *Enterococcus faecium*. However, after the development of diabetes, the bacterial flora in rat bulbar conjunctiva changed markedly, with reduced complexity in bacterial flora. Seven bacterial species, including *Enterococcus casseliflavus*, *Enterococcus cecorum*, *Enterococcus faecalis*, *Enterococcus saccharolyticus*, *Kocuria kristinae*, *Enterobacter aerogenes*, and *Proteus vulgaris*, emerged and five species, including *Klebsiella pneumoniae*, *Staphylococcus cohnii*, *Staphylococcus sciuri*, *Aerococcus viridans* and an unidentified species, disappeared.

**Table 1:** Bacterial species isolated from the conjunctiva of both STZ-induced diabetes and control groups.

Bacterial strain isolated	Diabetes group	Control group
*Enterococcus faecium*	23 (41.1%)*	14 (35.0%)
*Enterococcus gallinarum*	1 (1.8%)	4 (10.0%)
*Escherichia coli*	10 (17.9%)	4 (10.0%)
*Enterococcus casseliflavus*	1 (1.8%)	NI^#^
*Enterococcus cecorum*	1 (1.8%)	NI
*Enterococcus faecalis*	6 (10.7%)	NI
*Enterococcus saccharolyticus*	2 (3.6%)	NI
*Kocuria kristinae*	4 (7.1%)	NI
*Enterobacter aerogenes*	6 (10.7%)	NI
*Proteus vulgaris*	2 (3.6%)	NI
*Klebsiella pneumoniae*	NI	4 (10.0%)
*Staphylococcus cohnii*	NI	10 (25.0%)
*Staphylococcus sciuri*	NI	2 (5.0%)
*Aerococcus viridans*	NI	1 (2.5%)
Unidentified isolate	NI	1 (2.5%)
Total	56 (100%)	40 (100%)

* The numbers outside of brackets stand for the number of strain isolated for the corresponding species; the numbers in brackets represent the percentage of the bacterium in each group.

^#^ “NI” stands for “no isolation” of this bacterium in the corresponding group.

The most commonly isolated bacteria from both diabetic and healthy rats’ conjunctiva are Gram positive. As mentioned above, only three bacterial species were shared by both groups, and seven species emerged and five disappeared. Two species in the genus *Staphylococcus* are abundantly present in the conjunctiva of healthy rats, and are replaced by *Enterococcus* species and other bacterial species in diabetic rats. Enterococci exist abundantly in both human and animal feces and the changes in the bacterial species may reflect alterations in the microbiota in the intestines of diabetic rats [23]. The newly appeared bacteria in diabetic rats’ conjunctiva may cause pathological infections in the rats. For example, *E*. *casseliflavus* can cause endogenous endophthalmitis in humans [24]; *E*. *cecorum* is commonly isolated from poultry intestine and sometimes causes human infections [25]; and *K*. *kristinae* is also reported to be associated with diabetic foot and urinary tract infections [26, 27].

As far as we know, this is the first report on bacterial flora changes in rat conjunctiva after the induction of T1DM. The origin of the altered bacterial flora may be associated with the animal’s feces, skin or environment. However, the specific origin of the bacterial species should be investigated further. The results presented in this report have clinical implications and suggest that conjunctival bacterial flora in patients with diabetes should be surveyed for finding potential diagnostic markers or countermeasures to prevent eye infections in T1DM.
